# Effects of Probiotic (*Bifidobacterium longum* 35624) Supplementation on Exercise Performance, Immune Modulation, and Cognitive Outlook in Division I Female Swimmers

**DOI:** 10.3390/sports6040116

**Published:** 2018-10-10

**Authors:** Aaron F. Carbuhn, Shelby M. Reynolds, Clark W. Campbell, Luke A. Bradford, Jake A. Deckert, Andreas Kreutzer, Andrew C. Fry

**Affiliations:** 1 Department of Dietetics and Nutrition, University of Kansas Medical Center, Kansas City, KS 66160, USA; 2 Kansas Athletics, Inc., Lawrence, KS 66045, USA; reynolds@bronsonhg.org (S.M.R.); ccswim@ku.edu (C.W.C.); bradfordl@ku.edu (L.A.B.); 3 Osness Human Performance Laboratory, University of Kansas, Lawrence, KS 66045, USA; jaked6@ku.edu (J.A.D.); acfry@ku.edu (A.C.F.); 4 Department of Kinesiology, Texas Christian University, Fort Worth, TX 76129, USA; a.kreutzer@tcu.edu

**Keywords:** probiotic, swimmers, exercise, performance, inflammation, cognitive

## Abstract

Our aim was to determine the effects of probiotic supplementation (*Bifidobacterium longum* 35624; 1 billion CFU·d^−1^) on exercise performance, immune modulation, and cognitive outlook in collegiate female athletes during six weeks of offseason training. Seventeen National Collegiate Athletic Association (NCAA) Division 1 collegiate female swimmers participated in this two-group matched, double-blind, placebo controlled design. Via stratified randomization, participants were assigned to probiotic (*B. longum* 35624; *n* = 8) or placebo (*n* = 9) groups. Pre, mid, and post-training, all participants completed exercise performance testing (aerobic/anaerobic swim time trials and force plate vertical jump) as well as provided serum (cytokine and gastrointestinal inflammatory markers) and salivary immunoglobulin A samples. Recovery-stress questionnaire for athletes (RESTQ-Sport) was administered at baseline and conclusion of each week. Data were analyzed by analysis of covariance (ANCOVA) by time point with the respective baseline values of each dependent variable being the covariate. No significant differences in exercise performance and biochemical markers were observed between groups following offseason training. Recovery-Stress Questionnaire for Athletes (RESTQ-sport) values in *B. longum* 35624 group had significantly higher (i.e., more desired; *p* < 0.05) values in sport recovery (weeks five and six) than placebo. Probiotic supplementation in collegiate female swimmers did not affect exercise performance or immune function throughout offseason training, but did indicate alterations in cognitive outlook.

## 1. Introduction

The human gut microbiota contains a diverse bacterial species understood to exert numerous physiological functions such as protection against pathogens, barrier effects, regulation of energy levels and metabolism, modulation of intestinal motility, and regulation of immunity within the entire gastrointestinal (GI) tract [1]. Probiotic bacteria are defined as live microorganisms that confer a health benefit on the host when administered in adequate amounts [2]. Some specific probiotics/probiotic strains have been shown to beneficially impact the intestinal mucosal barrier and improve GI integrity, and thus, in turn, strengthening GI immune response, reducing mucosal inflammation, and decreasing oxidative stress [3,4,5,6,7,8].

Recent findings have shown a specific probiotic strain termed *Bifidobacterium longum* 35624 (*B. longum* 35624), a specific strain of Bifidobacterium, to beneficially impact the immune response by inducing regulatory T cells (T_reg_), which are designated to better contain reactions and limit the aggressive spread of immune responses to other tissues, in human models within the gut as well as beyond the gut [9,10,11]. It has been previously reported that six–eight weeks of *B. longum* 35624 supplementation increases secretion of T_reg_ cells in the peripheral blood of healthy human subjects as well as reduce systemic circulation of pro-inflammatory biomarkers TNF-α and IL-6 in clinical patients diagnosed with chronic fatigue syndrome (CFS) an immune-inflammatory disease [9,12].

In addition to these systemic immunomodulatory effects, it has also been demonstrated that increased concentrations of *Bifidobacterium* within the gut, through supplementation of *Lactobacillus casei* strain *Shirota*, can positively affect emotional symptoms in CFS patients such as reducing levels of anxiety and improving mood [13]. Moreover, chronic *B. longum* 35624 supplementation in the animal model has been found to positively influence neuronal systems and behaviors pertinent to depression [14,15]. These findings suggest some strains of *Bifidobacteria*, including *B. longum* 35624, could play a role in gut-brain interactions and provide, through supplementation, a prospective therapeutic application for these probiotics in response to various forms of cognitive stress.

In sports and exercise, it is common for highly trained athletes such as elite swimmers to undergo periods (>one week) of intensive exercise training made up of repetitive, high volume, intense concentrated workloads combined with inadequate periods of physical rest [16]. This particular exercise training regimen has been reported to result in an increased susceptibility of upper respiratory tract infections, GI symptoms (i.e., nausea, cramps, vomiting), physical exhaustion (i.e., fatigue), cognitive stress, and overall reductions in sport performance [17,18,19,20,21,22]. These potential undesired outcomes during this intensive exercise training phase are hypothesized to occur from increased microtrauma (i.e., injury) to the muscle, GI tract, connective tissue, and joints [23,24]. As a result, these injuries are proposed to initiate a local and “whole body” systemic inflammatory response that could be attributed to a compromise in immune function, decreased exercise performance, and negatively alter mood states [23].

Thus, the systemic immunomodulatory and cognitive effects observed with some strains of Bifidobacterium and more importantly *B. longum* 35624 supplementation in CFS patients could be of benefit in athletes like swimmers who participate in chronic, prolonged intensified exercise training resulting in potential changes to the GI tract, immune function, physical readiness, and cognitive stress. Therefore, the purpose of this study was to explore the effects of *B. longum* 35624 supplementation in swimmers with respect to exercise performance, immune modulation, and cognitive outlook during an intensive six-week exercise training phase.

## 2. Material and Methods

### 2.1. General Experimental Design

To determine the effects of *B. longum* 35624 on systemic inflammatory response, exercise performance, and cognitive stress during an intensified exercise-training phase, a double-blind, randomized placebo controlled analysis of covariance (ANCOVA) by time point design was utilized. Table 1 illustrates the general research design and procedures. The dependent variables for this study included markers of inflammation and immune function, multiple measures of exercise performance, as well as a weekly assessment of cognitive stress-recovery. Participants who met the inclusion criteria were randomly assigned, via stratified randomization, to ingest either *B. longum* 35624 or placebo. Supplementation commenced at the onset of designated intensified training phase thus requiring baseline measurements to be completed at least 72 h prior to beginning of the study and included all blood and salivary samples, exercise performance testing, and cognitive stress-recovery assessment. Additional blood/salivary samples and exercise testing to determine possible inflammation, immune, and performance variations during respective training phase were measured again at midpoint (week 3) and immediately upon completion (week 6) of said phase with the cognitive stress assessment being completed weekly (weeks 1–6).

### 2.2. Participants

Twenty (*n* = 20) Division 1 collegiate female swimmers were recruited to participate in this study. Participants are considered “high level” competitive swimmers with extensive swimming experience. These collegiate female swimmers were recruited by the University and provided athletic scholarships to compete for the University at the Division 1 level. All inclusion/exclusion criteria followed the Groeger et al. study (2013) using *B. longum* 35624 supplementation in CFS patients which states any participants pregnant or diagnosed with a lactose intolerance and immunodeficiency in addition to any previous abdominal surgery (with the exception of hernia repair or appendectomy) or reported psychiatric illness were excluded from the study [9]. Additionally, all recruited participants were required to refrain from taking any anti-inflammatory medications (Prescription and Non-prescription) or antibiotics for the previous month as well as throughout the duration of the six-week study. Finally, all participants must also report not having any nutritional supplements (with the exception of a multi-vitamin, vitamin C, vitamin D, and iron supplements) and ergogenic aids for the preceding one-week period as well as throughout the study. All participants were informed of the study procedures and were required to provide written consent. The study was approved through the University’s Human Research Protection Program and Institutional Review Board.

### 2.3. Nutrition and Supplementation

One week prior to beginning supplementation and training, all participants received sport-specific nutrition education by a Registered Dietitian (RD) who is a Certified Specialist in Sports Dietetics (CSSD) addressing appropriate nutritional needs throughout duration of the study. Participants then completed a three day dietary food log prior to start of the study followed by two additional three day dietary food logs at midpoint and immediately upon completion of study. These recorded dietary food logs were analyzed to ensure nutritional habits remained similar as well as each log refraining from foods rich in probiotics (ex. Kefir) and caffeine that could potentially influence our findings during the course of supplementation and exercise training.

Participants began the oral encapsulated supplementation regimen of either 1 capsule of 4 mg (1 × 10^9^ colony forming units (CFU) live bacteria) *B. longum* 35624 or an identical encapsulated placebo pill consisting of maltodextrin daily throughout their designated six week training phase. The entire respective supplementation dosage was ingested once each day. To ensure compliance, participants were blindly provided the supplement each day of practice and were visually observed ingesting their respective supplement by a member of the investigative team followed by notation verifying ingestion took place. On the day(s) participants were not required to train each participant received an individual supplement container enclosing their respective pill(s) and instructions to take as directed and return the empty container as well as complete a weekly supplement adherence verification form to help ensure compliance.

### 2.4. Intensified Exercise Training Phase

The designated six week intensified exercise training phase was similar for each supplemented group and was coordinated/conducted by the participant’s team sport coach and strength and conditioning coach. Both respective coaches coordinated and conducted the participant’s exercise training program during the entire competitive season prior to onset of the study. All subjects received two weeks of physical recovery before the start of the study’s training phase, which occurred during the team’s off-season. Weeks 1–4 of the intensified exercise training included 5 days of training totaling 8 h of swim training in addition to two 30 min resistance training sessions with the accumulative swim practice volume accumulating around 20 km each week. Average daily overall swim distance was approximately 4 km with swim duration being 95 min. During weeks 5–6 the exercise training increased to 6 days of training totaling 20 h of swim training, which included within the 20 h, three 45 min resistance training sessions with the swim practice volume accumulating around 40 km each week. Average daily overall swim distance was approximately 6.67 km with swim duration being 135 min.

### 2.5. General Performance Testing

Participants underwent exercise performance testing at three separate times points (pre, mid, and post) during the designated supplementation and training phase. Testing included a vertical jump force plate test as well as an aerobic and anaerobic swim-specific performance assessments. The force plate was completed for all participants in the morning before the team’s resistance training session in accordance with a standing vertical jump movement to measure rate of eccentric force production (N/s), concentric force production (N/kg), and overall vertical jump height (meters), as per the team’s specific force plate jump protocol that was conducted monthly during the competitive season. The aerobic swim test was a 500 m freestyle time trial and the anaerobic swim test was a 100 m freestyle time trial. Each swim test was conducted in a 25 m training pool located in the team’s home natatorium. Both swim tests were standard assessments of exercise performance frequently conducted by the head coach during the competitive season.

### 2.6. Blood and Salivary Collection, Systemic and GI Inflammatory/Immune Markers

In accordance with the supplementation and exercise performance testing, blood and salivary samples were also obtained pre, mid, and post of the supplementation/intensified training experiment. At each respective time point 5 mL of blood was collected from each participant via venipuncture in the afternoon, at rest, and before afternoon exercise training occurred that day. To obtain serum samples, all collected blood was allowed to clot at room temperature then separated by refrigerated centrifugation and subsequently aliquoted and frozen at −80 °C for later analyses according to assay procedure requirements. These frozen serum samples were used to measure markers of systemic inflammation (IFN-γ, IL-1β, IL-1ra, IL-2, IL-4, IL-5, IL-6, IL-10, IL-13, IL-17F, IL-22, TNF-α) using a custom luminex magnetic bead-based human cytokine panels A&B (R&D Systems, Bio-Techne, Minneapolis, MN, USA). All samples for the cytokine assays were thawed once and analyzed by the same technician using a MAGPIX^®^ multiplex system (Luminex, Austin, TX, USA). Each respective cytokine panel’s internal standards and concentration controls for all analytes were detected by the MAGPIX^®^ system. All samples were analyzed in duplicate, with low, mid, and high control concentration values mean coefficients of variation of 0.07%, 7.6%, 0.4% for IFN-γ, 1.6%, 7.7%, 4.9% for IL-1β, 0.6%, 4.1%, 2.2% for IL-1ra, 3.6%, 0.1%, 0.9% for IL-2, 0%, 7.6%, 1.7% for IL-4, 3.9%, 7.4%, 6.1% for IL-5, 2%, 5%, 3.8% for IL-6, 2.1%, 1.7%, 5.4% for IL-10, 1.7%, 3.5%, 1.6% for IL-13, 1.9%, 13.9%, 8% for IL-17F, 2.1%, 2%, 2.8% for IL-22, and 0.5%, 6.9%, 4.2% for TNF-α. Markers of GI integrity (endotoxin/LPS and LPS Binding Protein (LBP)) were measured using a pierce Limulus Amebocyte Lysate (LAL) chromogenic endotoxin quantitation kit (Thermo Scientific, Waltham, MA, USA) and human LBP duoset enzyme-linked immunosorbent assay (ELISA) (R and D Systems, Bio-Techne, Minneapolis, MN, USA). 

Salivary samples using Salimetrics SalivaBio swabs were used to collect and measure immunoglobulin A (IgA) immediately following collection of blood/serum samples. However, before participants provided a sample, per Salimetrics instructions, they were required to thoroughly rinse out their mouth with tap water and wait 10 min before providing a salivary sample. Once ready to provide a sample, participants were instructed to remove the SalivaBio Oral Swab (SOS) from packaging and place, without touching, in their mouth and under the tongue. Participants were required to keep the SOS under their tongue for two minutes to ensure it was fully saturated. After two minutes and full saturation, participants placed the SOS, by mouth and without touching, into the swab storage basket insert located in the swab storage tube, which was then capped and frozen immediately at −80 °C to be later analyzed using a Salimetrics salivary secretory IgA indirect enzyme immunoassay kit.

### 2.7. Cognitive Stress-Recovery Assessment

A cognitive stress-recovery assessment using the Recovery-Stress Questionnaire for Athletes was utilized at baseline and at the end of each training week to determine the participant’s cognitive outlook during the intensified training study [22]. The RESTQ-Sport questionnaire utilized was the short version consisting of 52 items used to assess, in a multidimensional way, an athlete’s stress-recovery state during a specific training cycle. Each item answered in the RESTQ-52 Sport, which is based on a Likert-type scale with values ranging from 0 (never) to 6 (always), indicates how often an athlete participated in certain activities during the previous three days and nights. Once completed each week, these 52 items generate 19 specific stress-recovery scales, which are grouped into four categories; general stress, general recovery, sport stress, and sport recovery in efforts to reflect, quantitatively, each supplementation group’s subjective cognitive assessment of stress and recovery during the intensified exercise training load.

### 2.8. Statistical Analysis

All statistical analyses were performed using SPSS V.22 (Chicago, IL, USA) software. Study data were analyzed by ANCOVA by time point design with the respective baseline values of each dependent variable being the covariate. The dependent variables were defined as the blood and salivary inflammatory/immune markers, exercise performance tests, and RESTQ-52 Sport scale scores, while the independent variables were the supplemented vs non-supplemented groups. Data was considered statistically significant when the probability of type I error is ≤0.05. Dietary food logs were analyzed using a One-Way ANOVA. To determine statistical significance of ≤0.05 between groups, a robust test of equality of means using Welch/Brown-Forsythe statistics was utilized due to homogeneity of treatment sample size variances being violated. In addition, due to unequal variances, Dunnett’s C post hoc analysis was used.

## 3. Results

### 3.1. Study Population, Supplementation, and Nutrition

Seventeen of the twenty recruited participants completed the study. Two participants dropped out due to illness while the third subject dropped out due to quitting the team. Therefore, nine participants in the placebo group and eight participants in the *B. longum* 35624 group completed the six-week intensified exercise training study. Supplementation compliance throughout the study, which included weekends, was 96.94% in the placebo group and 97.81% in the *B. longum* 35624 group. Dietary food logs reported no statistical difference in total energy calories, carbohydrates, protein, and fat dietary intake between the groups throughout the study.

### 3.2. Swim and Force Plate Performance Testing

No statistical significant difference were observed between supplemented groups aerobic and anaerobic swim performance testing as well as their force plate power testing measuring concentric/eccentric force production and overall vertical jump height (Table 2).

### 3.3. Systemic Cytokine, GI, and Salivary IgA markers

**Figure 1:**
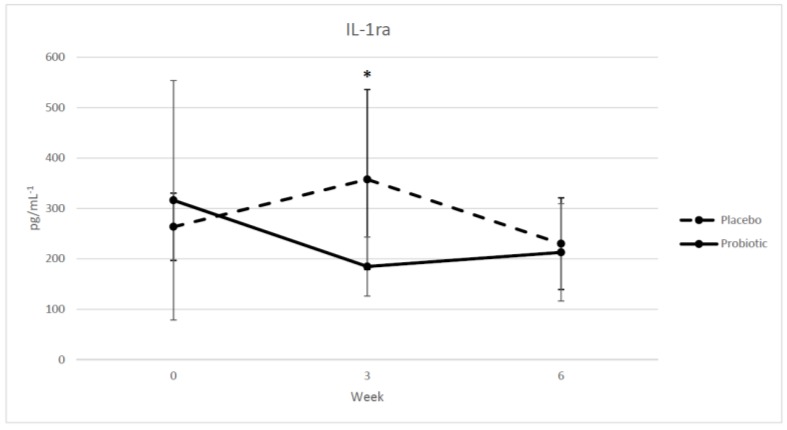
Serum concentrations of IL-1ra in collegiate female swimmers pre, mid, and post 6 weeks of exercise training and treatment. Probiotic the *B. longum* 35624 supplemented group, Placebo group, PRE week 0, MID week 3, POST week 6; *n* = 8 (*B. longum* 35624 supplementation), *n* = 9 (placebo). Values are means ± SD. There was a significant difference between groups at MID of exercise training and treatment: * *p* < 0.05 (ANCOVA).

**Figure 2:**
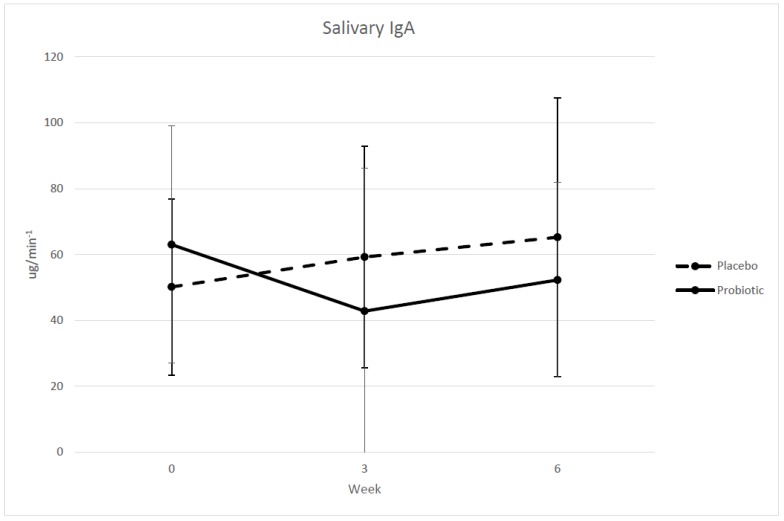
Salivary concentrations of IgA in collegiate female swimmers pre, mid, and post 6 weeks of exercise training and treatment. Probiotic the *B. longum* 35624 supplemented group, Placebo group, PRE week 0, MID week 3, POST week 6; *n* = 8 (*B. longum* 35624 supplementation), *n* = 9 (placebo). Values are means ± SD. *P*-value approached significance at MID of exercise training and treatment: *P* = 0.06 (ANCOVA).

Systemic inflammatory markers IFN-γ, IL-1β, IL-2, IL-4, IL-5, IL-6, IL-10, IL-13, IL-17, IL-17F, and IL-22, TNF-α were below detectable levels despite adhering to and meeting the luminex system’s quality assurance standards before and after each kit analysis. Each samples’ individual analyte mean bead count was > 150 and well above threshold for detection. Furthermore, each analyte’s standard curve r^2^ was > 0.992 and all low and mid controls were recovered for IL-1ra, IL-2, IL-4, IL-5, IL-6, IL-10, TNF-α, and IL-22. However, IL-13, IL-17F, and IGN- γ controls did not pass. Nonetheless, these lack of results indicate a potential the predetermined low concentration ranges from our designated kits were not sensitive enough in detecting systemic cytokine values for the majority of the measured inflammatory markers in a population of healthy athletes. Only IL-1ra in the *B. longum* 35624 group had statistically significantly lower (*p* = 0.029, η_p_^2^ = 0.296) serum levels at mid-training (week 3), in comparison to the placebo group (Figure 1). However, no statistical significant difference between groups in IL-1ra levels at post-training (week 6) was observed. Nevertheless, our measured IL-1ra concentration values were within range of previously published IL-1ra data reported in elite endurance athletes [25]. Serum GI markers endotoxin (LPS) and LBP were also not statistically significantly different between the supplementation groups. The *p*-value for salivary IgA levels approached significance with *B. longum* 35624 group measuring lower (*p* = 0.060, η_p_^2^ = 0.231) at mid-training in comparison to the placebo group (Figure 2). However, similar to IL-1ra levels, no significant difference was found between groups at post-training.

### 3.4. RESTQ-52 Sport

**Figure 3:**
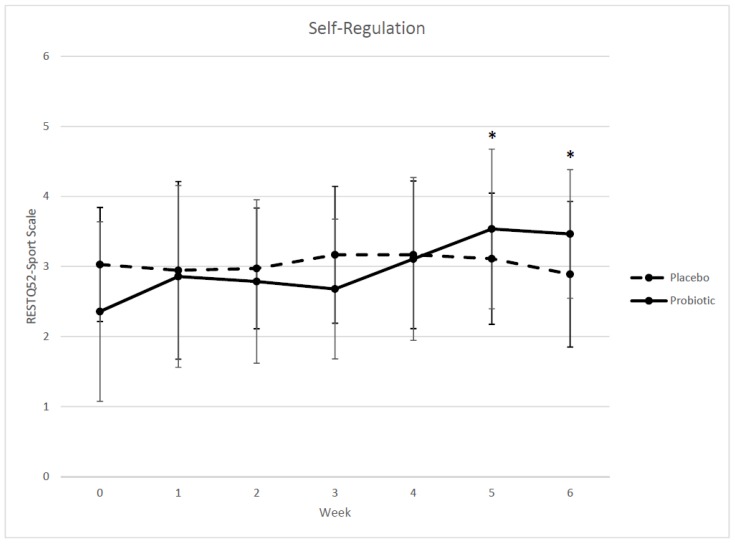
RESTQ52-Sport weekly self-regulation scores in collegiate female swimmers in 6 weeks of exercise training and treatment. The use of mental skills for athletes to prepare, push, motivate, and set goals for themselves are assessed by this scale. RESTQ52-Sport scale is defined as 0-never, 1-seldom, 2-sometimes, 3-often, 4-more often, 5-very often, 6-always. Probiotic the *B. longum* 35624 supplemented group, placebo group, week 0–6; *n* = 7 (*B. longum* 35624 supplementation), *n* = 9 (placebo). Values are means ± SD. There was a significant difference between groups at Week 5 and Week 6 of exercise training and treatment: * *p* < 0.05 (ANCOVA).

Table 3 shows the summary scores in each stress-recovery scale of the RESTQ-52 Sport for all seven measurements (weeks 0–6) taken during the study. During the first four weeks, which encompassed the 8 h training schedule, no statistical significant difference was observed between the groups until weeks three and four with significant changes noted within the “General Stress” category under the Conflicts/pressure scale and Social stress scale, respectively. The *B. longum* 35624 supplemented group reported during week three of training significantly higher values of Conflicts/pressure in comparison to placebo group. Following the conclusion of week four, *B. longum* 35624 group had significantly lower values in Social stress in comparison to placebo group. During the last two weeks of the study, which required both groups to undergo the 20 h exercise-training regime, a statistical significant difference was found within the “Sport Recovery” category under the Personal accomplishment scale (week five) and Self-regulation scale (weeks five and six). The B. longum 35624 group recorded significantly higher, more favorable, values in Self-regulation (Figure 3) scales, weeks five and six, in comparison to the placebo group.

## 4. Discussion

To the author’s knowledge, this was the first study to investigate *B. longum* 35624 supplementation in healthy collegiate athletes. More specifically, this study examined the effects of *B. longum* 35624 supplementation on exercise performance, immune modulation, and cognitive stress-recovery outlook in Division 1 collegiate female swimmers during a six week intensified exercise training phase. We did not observe any exercise performance differences between the supplemented group’s aerobic and anaerobic swim time trials as well as their force plate vertical jumps throughout the study. Similar to our findings, previous studies have also been unable to demonstrate probiotic supplementation’s ability to directly affect exercise performance [20]. Rather, probiotics effects on exercise performance has been suggested to be more indirect, as reported by Salarkia et al. 2013 using probiotic yogurt in young adult female endurance swimmers [26]. The probiotic yogurt contained several probiotic species (*Lactobacillus acidophilus*, *Lactobacillus delbrueckii bulgaricus*, *Bifidobacterium bifidum*, and *Streptococcus salivarus thermnophilus*) with a total bacteria count of 4 × 10^10^ CFU and was consumed for eight weeks. They found swimmers supplemented with the probiotic yogurt had significant improvements in VO_2max_. These VO_2max_ improvements were suggested to be related to reductions in the number of occurrences of respiratory infections in addition to the probiotic yogurt supplemented swimmers being healthier overall during the eight weeks of training. As a result of the yogurt group reporting an enhanced immune function and a reduced susceptibility to illness they suggest the probiotics from the yogurt indirectly allowed these swimmers to maximize their training benefits during the eight weeks of intensive exercise training resulting in a significant increase in VO_2max_.

In the present study, all participants who completed the six week exercise training were not diagnosed, by the team’s physician or physician’s assistant, with any respiratory infections or illnesses that kept them out from participation in the study. The two participants diagnosed with an illness and unable to train for more than several days were removed from the study and not included in the analysis. Since we were unable to find any differences in our exercise performance testing throughout the study and did not observe any changes between the groups’ occurrences of respiratory infections, or even trends reflecting the probiotic group to be less susceptible to illness, our findings were unable to support a potential indirect effect on exercise performance from probiotic supplementation as suggested by Salarkia et al. 2013 [26].

According to the results of our study, only a significant decrease in the systemic cytokine marker IL-1ra within the probiotic group at mid-training was found. IL-1ra is an anti-inflammatory cytokine and is a natural antagonist that competes with IL-1β, a pro-inflammatory cytokine, for receptor binding without initiating a pro-inflammatory signal transduction [27,28]. Significant elevations in IL-1ra levels of up to 40 fold increases have been reported to occur following most endurance exercise [29]. Research suggests this increase following endurance exercise is in response to the rapid increase of systemic IL-6 concentrations [30,31]. Since IL-1ra originates from mononuclear and polymorphonuclear leukocytes, as opposed to IL-6, which is produced locally from the working muscle during exercise, it is believed the leukocyte derived IL-1ra production is significantly increased when the pro-inflammatory production of IL-6 within skeletal muscle gets released in efforts to exert strong anti-inflammatory effects [31,32,33,34,35]. Thus, it is suggested that the levels of systemic IL-1ra reflect the production of IL-6 [25]. Unfortunately, we were unable to detect and measure systemic levels of IL-6. Therefore, we are unclear as to why IL-1ra levels during the mid-point of training were lower than placebo. However, given previous findings by Groeger et al. 2013, which demonstrates reduced systemic levels of IL-6 in CFS patients with *B. longum* 35624 supplementation, it could be speculated that similar effects occurred in our probiotic supplemented group’s systemic levels of IL-6 during the eight h exercise training regimen [9]. As a result of these “theorized” lowered IL-6 levels during and following exercise, it is plausible IL-1ra followed a similar pattern with systemic levels being lower after exercise and at rest in comparison to the placebo group.

As previously stated, we were unable to detect or even extrapolate the additional cytokines and their levels despite obtaining a high bead count for each marker during analysis. A previous study has reported IL-1β and IL-5 to be below detectable limits in the serum of healthy subjects [36]. However, cytokines IFN-γ, IL-2, IL-4, IL-6, IL-10, IL-13, IL-17, IL-17F, and TNF-α have been found to be detectable with referenced normative levels in the serum of healthy subjects [36]. Therefore, our inability to detect these previously referenced cytokines could be attributed to our designated cytokine kits not being sensitive enough to detect resting (i.e., outside of physical exercise) serum cytokine levels in healthy participants such as highly trained collegiate athletes.

To the author’s knowledge, this is the first study to examine the effects of probiotic supplementation on salivary IgA in swimmers during an intensive exercise training phase. No statistical significant difference in salivary IgA was observed between groups throughout the study. IgA is the predominant protein in the mucosal antibody response and is critical in helping neutralize toxins and remove pathogens [37]. Previous studies have shown chronic, prolonged intensive exercise to decrease salivary IgA levels and subsequently increase likelihood of developing an upper respiratory tract infection in athletes [18,38]. This inverse relationship between salivary IgA and development of upper respiratory tract infection has been reported to occur in marathon runners [39] with salivary IgA secretion being reduced by 10% followed by 25% of the runners reporting an upper respiratory tract infection within two weeks after completing the race. However, as previously stated, there were no respiratory infections or illnesses amongst any of our participants in either group which further support our salivary IgA results. Therefore, we did not observe a similar inverse relationship.

Gut inflammatory markers, endotoxin (LPS), and LBP were not significantly different between the two groups throughout the study. To our knowledge this is the first time both GI markers have been measured in swimmers undergoing an intensive exercise training phase. Our endotoxin levels measured amongst our group of collegiate swimmers were similar to levels reported by Lira et al. 2010 in highly trained cyclists [40]. Lira et al. 2010 found, and concluded, that endotoxin levels correlate negatively with highly trained subjects. Since our subjects are also highly trained we observed a similar correlation despite exercise training being strenuous and intensive. As a result of our endotoxin findings it was not surprising for LBP levels to remain unchanged between both the probiotic and placebo groups. LBP has been previously shown to play an important role in the inflammatory response, which is secondary to increased blood levels of endotoxin [41,42]. Since we observed no change, or difference, in serum endotoxin levels between groups throughout the study then it would almost expected for LBP levels to follow a similar pattern.

Our cognitive RESTQ52-Sport findings suggest the 8 h exercise training regimen (weeks one–four) was not a high enough exercise training load to illicit changes within the reported scales of general stress, general recovery, sport stress, and sport recovery. During those respective weeks we only found a couple of single time points (i.e., one week) differences between the two supplemented groups and lacked any repeated weekly differences to more clearly indicate a potential stress-recovery effect from both the training load and/or supplementation. A similar finding in regards to training load in swimmers using the RESTQ-Sport has been reported [21]. Gonzalez-Boto et al. 2008 also did not observe any differences within the swimmer’s stress-recovery scales during exercise training phases that involved overall swim distances ranging between 3.2 km–3.9 km and total swim durations of each session between 87–102 min. Interestingly, our 8 h exercise training week averaged a daily overall swim distance of 4 km and swim duration of 95 min. Therefore, it appears our weeks one–four exercise training were similar to Gonzalez-Boto et al. 2008 which supports our similar lack in the findings.

When our participants transitioned to the 20 h week exercise training phase (weeks five and six), which included daily overall swim distance of 6.67 km and swim duration of 135 min we observed the probiotic group to have significantly higher values in both respective weeks under the scale of self-regulation, which is part of the sport recovery group. The self-regulation scale is defined as the use of mental skills for athletes to prepare, push, motivate, and set goals for themselves [22]. Therefore, while undergoing a significantly higher exercise training load this cognitive scale suggest the probiotic supplemented group felt they were able to mentally push and motivate themselves more than the placebo group. Gonzalez-Boto et al. 2008 reported significant reductions in their swimmer’s sport recovery (being in shape, self-efficacy), sport stress (emotional exhaustion, injury), and general recovery (physical recovery) when the overall daily swim distance was five km and the overall swim duration was 137 min. Therefore, this finding suggests if *B. longum* 35624 supplementation was extended during a prolonged (ex. four–six weeks) 20 h exercise training load it is plausible the probiotic group would maintain a higher level of self-regulation. As a result, in theory, it could provide an indirect means of enhancing exercise performance based simply upon the probiotic group’s improved cognitive stress-recovery outlook.

## 5. Conclusions

This was the first study to investigate the effects of *B. longum* 35624 probiotic supplementation in athletes such as collegiate female swimmers. Our findings indicated that daily *B. longum* 35624 supplementation during a six-week exercise training phase did not directly influence exercise performance. However, the probiotic group reported higher sport recovery during the final two weeks of the offseason training program. These findings suggest the potential for an indirect effect on exercise performance if supplementation duration was extended and/or perhaps *B. longum* 35624 dosage altered. In regards to dosage, the Groeger et al. 2013 study provided for its CFS patients a *B. longum* 35624 daily dosage of 1 × 10^10^ CFU for six–eight weeks and reported significant systemic reductions in IL-6 and TNF-α [9]. Our daily dosage was 1/10 of the Groeger et al. 2013 study at 1 × 10^9^ CFU. Therefore, it would be of interest to investigate if a higher dosage in an athletic population could influence systemic inflammation and immune function as well as cognitive outlook throughout an extended higher exercise training load and provide an indirect exercise performance benefit. Thus, additional research is recommended to further investigate these duration and dosage questions in the hopes of better understanding potential indirect effects *B. longum* 35624 supplementation might have on the health and performance of athletes.

## Figures and Tables

**Table 1:** General research procedures.

		Week 0	Weeks 1 & 2	Week 3	Week 4	Week 5	Week 6
		Baseline	8 h	8 h	8 h	20 h	20 h
Monday	AM	-	Week 1	Week 2	Week 3	Week 4	Week 5
	PM	-	Compliance	Compliance	Compliance	Compliance	Compliance
Tuesday	AM	-	-	-	-	-	-
	PM	100 m Swim	-	100 m Swim	-	-	100 m Swim
Wednesday	AM	Force Plate	-	Force Plate	-	-	Force Plate
	PM	Blood sample	-	Blood sample	-	-	Blood sample
Thursday	AM	-	-	-	-	-	-
	PM	500 m Swim	-	500 m Swim	-	-	500 m Swim
Friday	AM	Return Logs	-	Return Logs	-	-	Return Logs
	PM	RESTQ52	RESTQ52	RESTQ52	RESTQ52	RESTQ52	RESTQ52

**Table 2:** Swim exercise performance and force plate vertical jump.

Performance	Week 0		Week 3		Week 6	
Test	Probiotic	Placebo	Probiotic	Placebo	Probiotic	Placebo
500 m Freestyle Aerobic	447 ± 21.9	457 ± 23.9	439 ± 15.8	443 ± 23.8	437 ± 18.6	443 ± 18.5
Swim Time Trial (sec)	(415–486)	(423–503)	(414–460)	(417–497)	(413–473)	(420–480)
100 m Freestyle Anaerobic	61.2 ± 2.2	64.8 ± 2.2	61.7 ± 1.8	63.1 ± 2.1	61.3 ± 2.2	63.8 ± 2.1
Swim Time Trial (sec)	(58.9–64.5)	(60.5–67.2)	(59.3–64.1)	(60.1–65.3)	(58.3–64.2)	(60.6–68)
Eccentric Force Production	4196.8 ± 1694.1	2842.8 ± 1298.2	4034.8 ± 1671.4	2565.3 ± 1458.5	3669.4 ± 1349.1	2619.4 ± 1500.7
(N/s)	(2130.7–6402.4)	(900–4732.5)	(1520.6–6148.1)	(870.7–5724.6)	(1642.9–5411.2)	(749.6–5282)
Concentric Force Production	18.15 ± 1.1	17.56 ± 1.79	17.96 ± 1.44	17.01 ± 1.7	17.83 ± 1.1	17.08 ± 1.79
(N/kg)	(16.68–20.13)	(15.53–20.65)	(15.71–20.09)	(15.24–20.71)	(16.3–19.69)	(15.33–21.35)
Vertical Jump Height	0.35 ± 0.05	0.34 ± 0.08	0.35 ± 0.06	0.32 ± 0.06	0.35 ± 0.05	0.32 ± 0.05
(m)	(0.25–0.43)	(0.28–0.54)	(0.26–0.41)	(0.29–0.48)	(0.28–0.42)	(0.29–0.44)

Values are mean ± SD, (Min–Max).

**Table 3:** RESTQ52-Sport cognitive stress-recovery.

RESTQ-Sport	Week 0		Week 3		Week 4		Week 5		Week 6	
Scale	Probiotic	Placebo	Probiotic	Placebo	Probiotic	Placebo	Probiotic	Placebo	Probiotic	Placebo
General Stress	-	-	-	-	-	-	-	-	-	-
1. General Stress	1.71 ± 0.48	1.61 ± 0.82	1.71 ± 1.1	1.72 ± 0.90	0.93 ± 0.67	1.28 ± 0.76	1.64 ± 1.3	1.94 ± 1.4	2.29 ± 0.99	2.44 ± 1.1
2. Emotional Stress	1.58 ± 0.58	1.78 ± 0.67	1.83 ± 0.93	2.00 ± 1.1	1.08 ± 0.66	1.78 ± 0.62	1.75 ± 0.94	2.1 ± 1.11	2.5 ± 1.1	2.44 ± 0.68
3. Social Stress	1.50 ± 0.87	1.61 ± 0.55	1.64 ± 0.85	1.67 ± 0.61	1.00 ± 0.65 *	1.72 ± 0.51	1.86 ± 0.99	2.00 ± 1.2	2.43 ± 1.1	2.33 ± 1.0
4. Conflicts/pressure	1.75 ± 0.82	2.44 ± 1.1	2.08 ± 0.97 *	1.67 ± 1.1	1.75 ± 0.99	2.05 ± 1.3	2.25 ± 1.4	2.22 ± 1.0	3.00 ± 1.1	2.56 ± 1.3
5. Fatigue	2.00 ± 1.0	1.89 ± 1.3	2.17 ± 0.93	1.94 ± 1.4	1.42 ± 1.7	1.72 ± 0.44	2.25 ± 1.7	2.33 ± 0.75	3.33 ± 1.6	2.44 ± 1.1
6. Lack of energy	1.92 ± 0.92	2.22 ± 0.91	2.50 ± 1.2	1.67 ± 0.90	1.58 ± 1.1	1.83 ± 0.50	1.25 ± 1.0	1.78 ± 0.83	2.25 ± 1.2	2.28 ± 0.87
7. Physical complaints	0.75 ± 0.52	1.39 ± 0.60	1.42 ± 1.1	1.56 ± 0.53	0.83 ± 0.82	1.22 ± 0.67	1.67 ± 1.3	1.50 ± 0.79	2.00 ± 0.89	1.55 ± 0.77
General Recovery	-	-	-	-	-	-	-	-	-	-
8. Success	2.00 ± 0.63	3.28 ± 0.83	2.17 ± 0.75	2.61 ± 0.74	2.67 ± 0.93	3.17 ± 1.1	3.25 ± 1.5	2.78 ± 1.1	2.75 ± 1.1	3.11 ± 1.1
9. Social recovery	3.42 ± 0.66	4.28 ± 0.67	2.67 ± 0.93	3.67 ± 1.0	3.50 ± 0.32	3.89 ± 0.70	3.50 ± 0.89	3.56 ± 1.2	3.33 ± 1.2	3.50 ± 1.3
10. Physical recovery	1.67 ± 0.82	2.44 ± 1.0	2.00 ± 0.77	2.33 ± 0.79	2.42 ± 0.97	2.61 ± 0.89	2.75 ± 1.2	2.50 ± 0.75	1.83 ± 0.93	2.39 ± 0.82
11. General well-being	2.75 ± 1.3	3.94 ± 0.68	2.83 ± 1.6	3.17 ± 1.2	3.75 ± 0.42	3.67 ± 0.97	3.00 ± 1.4	3.22 ± 1.1	2.83 ± 1.1	3.17 ± 1.3
12. Sleep quality	3.50 ± 1.3	3.78 ± 0.67	3.86 ± 0.56	3.89 ± 0.65	4.07 ± 0.89	3.72 ± 0.75	4.14 ± 0.75	3.67 ± 0.79	3.36 ± 0.99	3.50 ± 1.0
Sport Stress	-	-	-	-	-	-	-	-	-	-
13. Disturbed breaks	0.96 ± 0.39	1.25 ± 0.80	1.04 ± 0.57	1.25 ± 0.73	1.04 ± 0.83	1.25 ± 0.72	1.07 ± 0.66	1.56 ± 0.60	2.04 ± 1.3	1.86 ± 1.1
14. Emotional exhaustion	1.64 ± 0.91	1.75 ± 0.94	1.64 ± 0.35	1.78 ± 1.3	1.14 ± 0.75	1.47 ± 1.1	1.69 ± 1.3	1.81 ± 0.93	1.68 ± 1.3	2.25 ± 0.93
15. Injury	2.32 ± 0.88	1.86 ± 1.2	2.89 ± 0.93	2.56 ± 1.4	2.14 ± 0.75	2.19 ± 1.1	3.04 ± 1.2	2.64 ± 0.82	3.29 ± 1.2	2.19 ± 0.87
Sport Recovery	-	-	-	-	-	-	-	-	-	-
16. Being in shape	1.75 ± 1.1	2.58 ± 0.94	2.14 ± 0.63	2.92 ± 1.4	2.75 ± 0.60	3.39 ± 1.0	2.68 ± 1.1	2.92 ± 0.94	2.54 ± 1.1	2.78 ± 1.1
17. Personal accomplishment	2.54 ± 1.0	3.14 ± 0.49	2.57 ± 1.1	3.31 ± 0.99	3.32 ± 0.93	3.14 ± 0.95	3.43 ± 0.93 *	2.75 ± 0.87	2.79 ± 1.2	2.72 ± 1.1
18. Self-efficacy	1.89 ± 1.1	2.64 ± 0.87	2.43 ± 0.70	3.47 ± 1.1	3.07 ± 0.79	3.44 ± 0.92	3.25 ± 1.1	3.00 ± 1.0	3.08 ± 1.2	3.08 ± 1.2
19. Self-regulation	2.35 ± 1.3	3.03 ± 0.81	2.68 ± 1.0	3.17 ± 0.98	3.11 ± 1.2	3.17 ± 1.1	3.54 ± 1.1 *	3.11 ± 0.94	3.46 ± 0.92 *	2.89 ± 1.0

Scores in the different scales of the RESTQ52-Sport corresponding to the seven measures (Week 0–6) taken during the training period. Values are mean ± SD. * *p* ≤ 0.05 significant difference between groups each week.
